# Ensuring on-time quality data management deliverables from global clinical data management teams

**DOI:** 10.4103/2229-3485.71774

**Published:** 2010

**Authors:** Zia Haque

**Affiliations:** *Clinical Data Management, Clinsys Clinical Research, Raleigh, NC, USA*

**Keywords:** Ontime delivery, quality, clinical data management

## Abstract

The growing emphasis on off-site and off-shore clinical data management activities mandates a paramount need for adequate solutions geared toward on-time, quality deliverables. The author has been leading large teams that have been involved in successful global clinical data management endeavors. While each study scenario is unique and has to be approached as such, there are several elements in defining strategy and team structure in global clinical data management that can be applied universally. In this article, key roles, practices, and high-level procedures are laid out as a road map to ensure success with the model.

## INTRODUCTION

Over the past several years, there has been a gradual shift in focus on the operational model for clinical data management activities in clinical research endeavors. The early trends tended to embrace a model wherein the clinical data management (and often times the related Biostatistics area of the trial) activities were conducted at offsite or off shore locations for studies involving investigative sites in the United States and Rest of the World (ROW). Cost advantages and the opportunity to harness the time difference were two significant factors in favor of this approach. India was the pioneer location in this avenue;[1] sustained successes in the software industry were distinct advantages in being able to potentially apply similar practices across the two industries.

The above described model was followed by an expanded approach which noticed a growing emphasis on the need for investigative sites in ROW locations. Spiraling costs associated with new drug development coupled with increasing competition for subject recruitment in competing trails meant that sponsors had to look at ROW site locations for solutions to these two areas. This development also meant that sponsors and Clinical Research Organizations (CRO) needed to address global clinical development efforts from the perspective of global data consistency.[2]

In looking at the present state of global clinical data management, it is a useful background to trace the history of how this operational piece has progressed over the past decade. Initial players who took the plunge to adopt this model were influenced by the successes of the software industry, which had experienced tremendous success with both off-site and off-shore models. It seemed logical that the successes from this model could be applied seamlessly into the clinical research arena, and the initial round of sponsors who decided to pursue this route were attracted by the significant cost advantages. It took perhaps up to 5 years for the industry to realize and acknowledge that this model needed to be revisited and revamped. Quality, timeline management, and communications seemed to be the top three areas that needed addressing at different levels within the model. The state of affairs at this juncture afforded a prime opportunity for CROs and sponsors to go back to the drawing board and chart out clearly defined expectations for processes to ensure success with the global clinical data management model.[3] A key differentiator between the software and clinical research industries is the fact that the former affords opportunities to be able to troubleshoot and fine tune the product to ultimate (or close to) perfection. With clinical research, however, there is but one window of opportunity to be able to design a pristine model for data collection. If this opportunity is not harnessed to perfection, the repercussions are often times irreversible and tend to manifest themselves with chronic regularity, impacting all deliverables downstream.

The author has been involved actively in studying and researching global clinical data management models with a view toward developing a fitting model to address the clear need for consistency in this operational area.[4] Active involvement with teams in the areas of project management, programming, and Biostatistics has helped understand and clarify expectations from internal teams in collaborating with global data management teams. In-depth involvement in the areas of business development support and client relationship management has also allowed the author to work with global data management teams to fashion the most appropriate model.

The paramount lesson in ensuring success in global clinical data management is perhaps acknowledging the fact that one size does not fit all! Often times there may be a temptation to create universal costing and resourcing models and apply these as solutions in response to all Requests for Proposals (RFP). Every RFP must be researched as a unique opportunity, and the temptation to force fit standard costing to all RFPs should be avoided. Ensuring active participation from key stakeholders at relevant global data management sites is a step in the correct direction. A global proposal owner should manage the progress of the proposal, and communicate actively with the global proposal team and other operational areas. Following the submission of the proposal, if appropriate relevant team members from the global data management location/s should form part of the bid defense team. This approach helps in demonstrating very early on to the prospective sponsor that the CRO is committed to the success of the study.

Assuming a project award following the bid defense of the proposal, the proposed global clinical data management team would progress from being a concept to reality. A global clinical data manager functions as the lightening rod for internal and external teams; the level of this role would be dictated by the volume and complexity of the study. Key areas of operational expertise that would be default requirements of this role would be strong project management experience, coupled with robust experience in client interaction and clear understanding of timelines for all the operational areas of the clinical trial, within and outside of clinical data management. The person in this role has to function with the clear understanding that they have a pivotal role in bridging the global data management team, other operational areas within their organization, as well as the sponsor. In scenarios where the global data management team is contracted to conduct only data management activities, there will be a larger number of outside teams to collaborate with.

Close attention to creating a well-defined communication plan forms the framework in which the global data management team can function with precision. While the global clinical data manager is the binding thread that holds the team in unison, regional lead data managers should be identified at all global locations. The team would function in a matrix model, wherein individual team members would continue to report to their line managers administratively, while reporting to the global clinical data manager operationally. A “Train the Trainer” approach is the best fit in global data management endeavors; the regional lead data managers would be trained on project-specific data management practices by the global clinical data manager and in turn would train the data managers at their location.

As mentioned earlier, project management skills are an integral part of the role of the global clinical data manager.[5] The acute need for project management within data management should be well understood by this role, especially in dealing with potential shifts in timelines, and in managing client expectations related to deliverables. The possibility of global data management teams being blindsided by timeline shifts is real, and proactive planning and communication from the global clinical data manager are critical in mitigating this risk.

Clear understanding of the nature of deliverables is another area where the global clinical data manager needs to establish clear understanding from internal and external teams. Some examples of this are as follows: what is the client expectation on any interim deliverable data transfers – are these transfers off of clean or “dirty” data? What is the frequency of sending out dictionary coding reports, and are these to be fully coded? Are resolutions to Data Clarification Forms (DCF) acceptable via faxed images? Does the client have specific formats for status reports? Focused discussions with the client around the entire spectrum of data management deliverables is of utmost importance; in addition to ensuring that mutual expectations on the extent and timing of deliverables is clearly established for the global teams, the exercise also helps in validating the Scope of Work (SOW) in the contract and can flag potential areas that may need to be addressed via a change order.

As the clinical trial gets under way, the global clinical data manager has the responsibility to monitor enrollment timelines, and communicate updates on when the data are expected in-house for processing. Ensuring accurate and on-time completion of all required data management documentation is another area that the global clinical data manager needs to coordinate closely with the global data management team. Rapid activity at different phases of the study poses a risk of study documentation not being completed on time, and proactive planning from the global lead data manager in ensuring timely reviews of applicable documentation within data management (by internal and external teams) followed by approval needs to be emphasized. Ongoing management of deliverables, active communication on any trends in data quality should be proactively managed by the global lead data manager.

A clear message that communicates the fact that the global data management team is expected to function as a smooth continuum, regardless of physical location, should be emphasized right across the study. This message should be emphasized across other internal operational areas. Any concerns with the global data management operations that the other (internal) operational teams may have should be discussed promptly, addressed at the appropriate level and followed to satisfactory closure. The role of the global lead data manager in cementing a tight working relationship between the other operational areas and the global data management teams is paramount and critical at the same time.

In scenarios where the model involves global clinical programming and Biostatistics teams working with global data management teams, the global clinical data manager can contribute a very effective value add role, in addition to the roles detailed above. Proactive communication with counterparts in these two operational areas is very helpful in ensuring that they can realign timelines and resources as required.

Recognizing and celebrating team achievements validates the importance of global contributions to team members at all locations. The global clinical data manager should ensure that project milestone achievements are acknowledged and celebrated. Global clinical data management represents exciting opportunities for success at individual and team levels; deliberate and proactive planning coupled with close communications will ensure accomplishment of quality and on-time deliverables by the global clinical data management team.
